# A Comparative Analysis of the Full and Short Versions of the Social Responsiveness Scale in Estimating an Established Autism Risk Factor Association in ECHO: Do we Get the Same Estimates?

**DOI:** 10.1007/s10803-023-06020-8

**Published:** 2023-07-22

**Authors:** Marisa A. Patti, Xuejuan Ning, Mina Hosseini, Lisa A. Croen, Robert M. Joseph, Margaret R. Karagas, Christine Ladd-Acosta, Rebecca Landa, Daniel S. Messinger, Craig J. Newschaffer, Ruby Nguyen, Sally Ozonoff, T. Michael O’Shea, Rebecca J. Schmidt, Cindy O. Trevino, Kristen Lyall

**Affiliations:** 1 A.J. Drexel Autism Institute, Drexel University, Philadelphia, PA USA; 2 Department of Epidemiology, Johns Hopkins Bloomberg School of Public Health, Baltimore, MD USA; 3 Wendy Klag Center for Autism and Developmental Disabilities, Johns Hopkins Bloomberg School of Public Health, Baltimore, MD USA; 4 Division of Research, Kaiser Permanente, Oakland, CA USA; 5 Department of Anatomy and Neurobiology, Boston University School of Medicine, Boston, MA USA; 6 Department of Epidemiology, Geisel School of Medicine, Dartmouth College, Hanover, NH USA; 7 Center for Autism and Related Disorders, Department of Psychiatry and Behavioral Sciences, Kennedy Krieger Institute, Johns Hopkins University School of Medicine, Baltimore, MD USA; 8 Departments of Psychology and Pediatrics, University of Miami, Coral Gables, FL USA; 9 College of Health and Human Development, Pennsylvania State University, University Park, New York City, PA USA; 10 Department of Epidemiology and Community Health, University of Minnesota, Minneapolis, MN USA; 11 Department of Psychiatry and Behavioral Sciences, MIND Institute, University of California Davis, Sacramento, CA USA; 12 Department of Pediatrics, University of North Carolina School of Medicine, Chapel Hill, NC USA; 13 Department of Public Health Sciences, UC Davis, UC Davis MIND Institute, Davis, Sacramento, CA, CA USA; 14 Department of Psychiatry and Behavioral Sciences, University of Washington, Seattle Children’s Research Institute, Seattle, WA USA

**Keywords:** Autism spectrum disorder, Qualitative traits, Social Responsiveness Scale, Social, Communication

## Abstract

**Purpose:**

Prior work developed a shortened 16-item version of the Social Responsiveness Scale (SRS), a quantitative measure of social communication and autism spectrum disorder (ASD)-related traits. However, its properties for use in risk factor estimation have not been fully tested compared to the full SRS. We compared the associations between gestational age (previously established risk factor for ASD) and the 65-item “full” and 16-item “short” versions of the SRS to test the shortened version’s ability to capture associations in epidemiologic analyses of ASD risk factors.

**Methods:**

We used data from participants in the Environmental influences on Child Health Outcomes (ECHO) Program (n = 2,760). SRS scores were collected via maternal/caregiver report when children were aged 2.5–18 years. We compared estimates of associations between gestational age and preterm birth between the full and short SRS using multivariable linear regression, quantile regression, and prediction methods.

**Results:**

Overall, associations based on full and short SRS scores were highly comparable. For example, we observed positive associations between preterm birth with both full ($\beta$=2.8; 95% CI [1.7, 4.0]) and short ($\beta$=2.9; 95% CI [1.6, 4.3]) SRS scores. Quantile regression analyses indicated similar direction and magnitude of associations across the distribution of SRS scores between gestational age with both short and full SRS scores.

**Conclusion:**

The comparability in estimates obtained for full and short SRS scores with an “established” ASD risk factor suggests ability of the shortened SRS in assessing associations with potential ASD-related risk factors and has implications for large-scale research studies seeking to reduce participant burden.

**Supplementary Information:**

The online version contains supplementary material available at 10.1007/s10803-023-06020-8.

Autism spectrum disorder (ASD), characterized by difficulties in social communication and restricted and repetitive behaviors, affects roughly 2% of 8-year-old children in the United States (U.S., Maenner et al., 2021). The diagnosis of ASD results in a binary categorization of having the condition or not; however, the continuum of the broader ASD-related phenotype extends beyond diagnostic boundaries into and through the general population (Billeci et al., 2016; Constantino & Todd, 2003, 2005; Robinson et al., 2016). The Social Responsiveness Scale (SRS, Constantino & Gruber 2005; Constantino et al., 2003; Constantino JN, Gruber C. 2012) is a 65-item informant-report tool and widely-used quantitative measure of the ASD-related phenotype (Bölte et al., 2011; Constantino et al., 2003). A 16-item shortened version of the SRS, derived from the full SRS, was proposed to abbreviate administration time and to reduce the potential influence of other psychiatric morbidities on ASD trait assessment (Sturm et al., 2017). This shortened SRS is increasingly being used to estimate risk factor associations. As a quantitative trait measure, the shortened SRS provides the opportunity to assess whether a given risk factor leads to a shift in a given trait across the entire population. However, this shortened version was developed in an autism-skewed sample, (Sturm et al., 2017) and its use as a quantitative trait measure for risk factor estimation has not been fully examined. Note that the term “risk factor” is used here as a statistical term and should not be interpreted as implying prevention of ASD related traits.

Prior work has evaluated the validity of the 16-item shortened SRS, and compared the psychometric properties in comparison to the full 65-item SRS (Kaat et al., 2023; Lyall et al., 2021, 2022; Sturm et al., 2017). However, limited prior work has addressed whether abbreviated versions of a neurodevelopmental quantitative trait measure capture risk factor associations in the same ways as their full counterparts. This is a key question to address given increasing use of abbreviated versions in large studies of child health (Gillman & Blaisdell, 2018; Hofman et al., 2004; Volkow et al., 2018).

Here we compare the extent to which an established risk factor for ASD — gestational age or preterm delivery — is associated with child full and short SRS scores in order to assess the shortened version’s ability to capture this relationship. We hypothesize that risk factor estimates between gestational age and preterm delivery will be similar between the shortened and full SRS, based on prior work observing comparability in psychometric properties between the full and short SRS (Kaat et al., 2023; Lyall et al., 2021, 2022; Sturm et al., 2017). We selected gestational age as a well-replicated factor associated with ASD (with preterm birth and low gestational age both consistently associated with increased odds of ASD, Gardener et al., 2011; Jenabi et al., 2021; Kuzniewicz et al., 2014; Mahoney et al., 2013) and compared estimates obtained for full and short SRS scores. We leveraged data from a large, geographically diverse sample of U.S. children from the Environmental influences on Child Health Outcomes (ECHO) program.

## Methods

### Study Sample

The study sample was drawn from the ECHO Program, a national consortium of 69 cohort studies investigating the effects of environmental exposures on child health (Gillman & Blaisdell, 2018; LeWinn et al., 2021). Individual cohorts follow common protocols for assessing child health and are overseen by single and site-specific institutional review boards. Participants provided informed consent for themselves and their children.

We analyzed data from 11 ECHO cohorts with SRS scores on children aged 2.5–18 years. Details of the 11 included cohorts and distributional properties of SRS scores have been previously described (Lyall et al., 2021). Briefly, among the 5,394 participants from 11 ECHO cohorts, 2,363 were excluded for missing child level information (i.e., age) or SRS scores. Of the 3,031 remaining participants, 271 were excluded for missing gestational age, resulting in a final analytic sample of 2,760 child-parent dyads.

### Social Responsiveness Scale (SRS)

Details of the SRS in ECHO cohorts have been previously described (Lyall et al., 2021). Briefly, the SRS is a 65-item questionnaire (referred to here as the “full” SRS) assessing autism-related traits, or social reciprocity, including social communication and restricted repetitive behaviors (Constantino & Gruber, 2005; Constantino et al., 2003; Constantino JN, Gruber C. 2012). Individual items are scored from 0 to 3 and summed to yield a total score (range: 0–195) where higher values indicate more ASD-related traits. Established thresholds reliably distinguish ASD children from both non-affected children and those with other conditions (e.g., intellectual disability, Constantino & Todd 2003). Additionally, cut-off scores (total raw score of $\ge$ 52 for the full SRS and $\ge$ 13 for the short SRS) are generally consistent with clinically relevant deficits in reciprocal social behaviors that may interfere with daily social interactions, and diagnosis of autism. In this sample, the full SRS (SRS or SRS-2) was completed by primary caregivers when children were 2.5–<18 years of age. Depending on child age, caregivers completed the preschool or school age forms (applicable to ages 2.5–4.5 or 4-<18 years, respectively). Of note, SRS versions are nearly identical, and only minor differences exist across forms to align with developmental relevance (i.e., age-appropriate examples).

The 16-item SRS (hereafter referred to as the “short score”) was developed based on item response theory (IRT) with the goal of increasing score efficiency (i.e., near-equivalent score precision in fewer items) and reducing participant burden along with reducing potential biases related to age, sex, and expressive language (Sturm et al., 2017). Using the existing 65-item SRS and data from autism registries, Sturm and colleagues developed the 16-item shortened version using item response theory to identify items from the existing 65-item full version. Item selection was based on high factor loadings, low evidence for differential functioning, and expert consideration of content validity. Short scores are calculated by summing the individual 16 items (range: 0–48); short scores in this analysis were calculated by summing only these 16 items abstracted from the 65-item administrations. Prior work has established the comparability of the shortened version of the SRS as a quantitative trait measure and in the prediction of ASD for use in screening purposes, similar to the full SRS (Lyall et al., 2021, 2022).

SRS scores are presented as raw scores due to lack of population-based T-score norms for short scores and minor norming differences across SRS forms (preschool and school age). We re-scaled scores (range: 0–100) using the percent of maximum possible method to enable direct comparison of full and short SRS scores. This scaling method has the advantage of reflecting group differences proportional to the original scale (Cohen et al., 1999). Note, we considered total scores in our analyses, not subscales, given that the short SRS does not allow for calculation of all subscales available with the full SRS.

### Model Risk Factor

Gestational age at birth in weeks was calculated based on maternal report of last menstrual period, as obtained via report on questionaries or in medical records, and child date of birth. We considered gestational age in weeks as a continuous measure as well as a categorical variable for preterm (defined as birth $<$37 weeks gestation).

### Statistical Analyses

First, we characterized distributions of study sample characteristics. Then, we compared covariate adjusted linear regression results between full and short SRS scores with gestational age and preterm birth. We adjusted for covariates based on *a priori* knowledge including maternal education, race/ethnicity, and child sex and age at the time when the SRS was administered. In addition, in order to assess whether the short SRS scores estimate associations across the full range of the score distribution in the same way as full SRS scores, we conducted quantile regression analyses (Beyerlein, 2014; Koenker, 2005; Koenker & Hallock, 2001). Compared to linear regression models that estimate mean differences in outcomes across distributions of exposures, quantile regression models allowed us to compare the direction and magnitude of associations between gestational age and the full and short SRS across quantiles of these scores. These analyses therefore address whether the exposure-outcome association is consistent or differs across the outcome distribution, as would indicate if the exposure shifts the entire trait distribution, or, say, just the upper tail of it.

In secondary analyses, we examined the ability of the full and short SRS to predict preterm birth (< 37 weeks gestation) as the *outcome*. In these models, rather than estimating influences on ASD-related traits associated with a known risk factor (i.e., gestational age), the goal was to compare the ability of the full and short scores to predict the known risk factor. These analyses were conducted with SRS scores parameterized continuously as well as categorically based on clinically relevant cut-off scores. We also conducted analyses stratified by source population, including those drawn from general population cohorts, preterm birth cohorts, and familial ASD enriched cohorts (selective enrollment of participants who previously had a child with ASD), to address whether differences in the trait distributions and likelihood of ASD in these populations influenced results.

We completed all statistical analyses using SAS Studio version 3.71 (SAS Institute, Inc. Cary, North Carolina). Figures were developed using R Studio version 4.1.1 (R Core Team, Vienna, Austria).

## Results

The analytic sample included 11 cohorts with n = 2,760 parent-child dyads. Basic characteristics of the study population are shown in Table 1. Approximately 22% of children were born preterm (with the prevalence increased over the general population rate owing to inclusion of preterm birth cohorts), with an average gestational age of 36.0 weeks (SD 5.1). The average age of SRS administration among children was 7.2 years (SD 5.1).

**Table 1:** Distribution of mother and child study, sociodemographic, and perinatal characteristics, among ECHO Cohorts (n = 2,760)

	N (%)
Cohort source population	
General population cohorts^a^	1,995 (72.3%)
Familial ASD enriched cohorts^b^	332 (12.0%)
Preterm birth cohorts^c^	433 (15.7%)
Maternal race/ethnicity	
Hispanic	238 (8.6%)
Non-Hispanic White	2,005 (72.6%)
Non-Hispanic Black	243 (8.8%)
Non-Hispanic other	175 (6.3%)
Missing	99 (3.6%)
Maternal education	
High school graduate or less	440 (15.9%)
Some college	521 (18.9%)
Bachelor’s degree or higher	1,736 (62.9%)
Missing	63 (2.3%)
Parity^d^	
No prior child	1,166 (42.3%)
≥ 1 prior child	1,471 (53.3%)
Missing	123 (4.5%)
Child sex	
Girl	1,337 (48.4%)
Boy	1,423 (51.6%)
Preterm^e^	
Yes	608 (22.0%)
No	2107 (76.3%)
Missing	45 (1.6%)
Prenatal health insurance	
Public	272 (9.9%)
Private	1,201 (43.5%)
Missing	1,266 (45.9%)
Prenatal vitamin use	
Yes	1,894 (68.6%)
No	89 (3.2%)
Missing	777 (28.2%)
Prenatal maternal smoking	
Yes	214 (7.8%)
No	2283 (82.7%)
Missing	263 (9.5%)

ECHO = Environmental Influences on Child Health Outcomes; ASD = Autism Spectrum Disorder.

^a^General population refers to ECHO cohorts drawn from the general population.

^b^Familial ASD enriched refers to ECHO cohorts with participants at high familial risk for ASD, due to selective enrollment of children whose mothers previously had a child diagnosed with ASD.

^c^Preterm birth refers to ECHO cohorts with selective enrollment of children born preterm.

^d^By study design, all participants in familial ASD enriched cohorts are multiparous.

^e^Preterm defined as birth < 37 weeks gestation. By study design, all participants in preterm birth cohorts are born preterm.

Overall, effect measures from regression models for associations with gestational age and preterm birth were similar between the full and short SRS. While continuous gestational age was not associated with full or short SRS scores in linear regression analyses, we observed positive associations of comparable magnitude between preterm birth and higher SRS scores based on the full ($\beta$ = 2.8; 95% CI [1.7, 4.0]) and short SRS ($\beta$ = 2.9; 95% CI [1.6, 4.3]) scores, (Table 2). Stratified by cohort type, associations estimated using the full and short SRS scores were also similar (Supplementary Table S1).

**Table 2:** Adjusted associations between gestational age and preterm birth with child SRS scores using the full and short SRS among ECHO Cohorts (n = 2,714)^a,b^

	Full SRS	Short SRS
Continuous GA^c^	-0.3 (-0.4, -0.2)	-0.3 (-0.4, -0.2)
Preterm birth^d^	2.8 (1.7, 4.0)	2.9 (1.6, 4.3)

ECHO = Environmental Influences on Child Health Outcomes; SRS = Social Responsiveness Scale; GA = Gestational Age

^a^Raw SRS scores were scaled from 0-100 to allow for comparison on the same scale

^b^Adjusted for maternal education, maternal race/ethnicity, child sex, age at SRS administration

^c^Continuous gestational age modeled as 1-week increase in gestational age

^d^Preterm birth is defined as < 37 weeks gestation

**Fig. 1:**
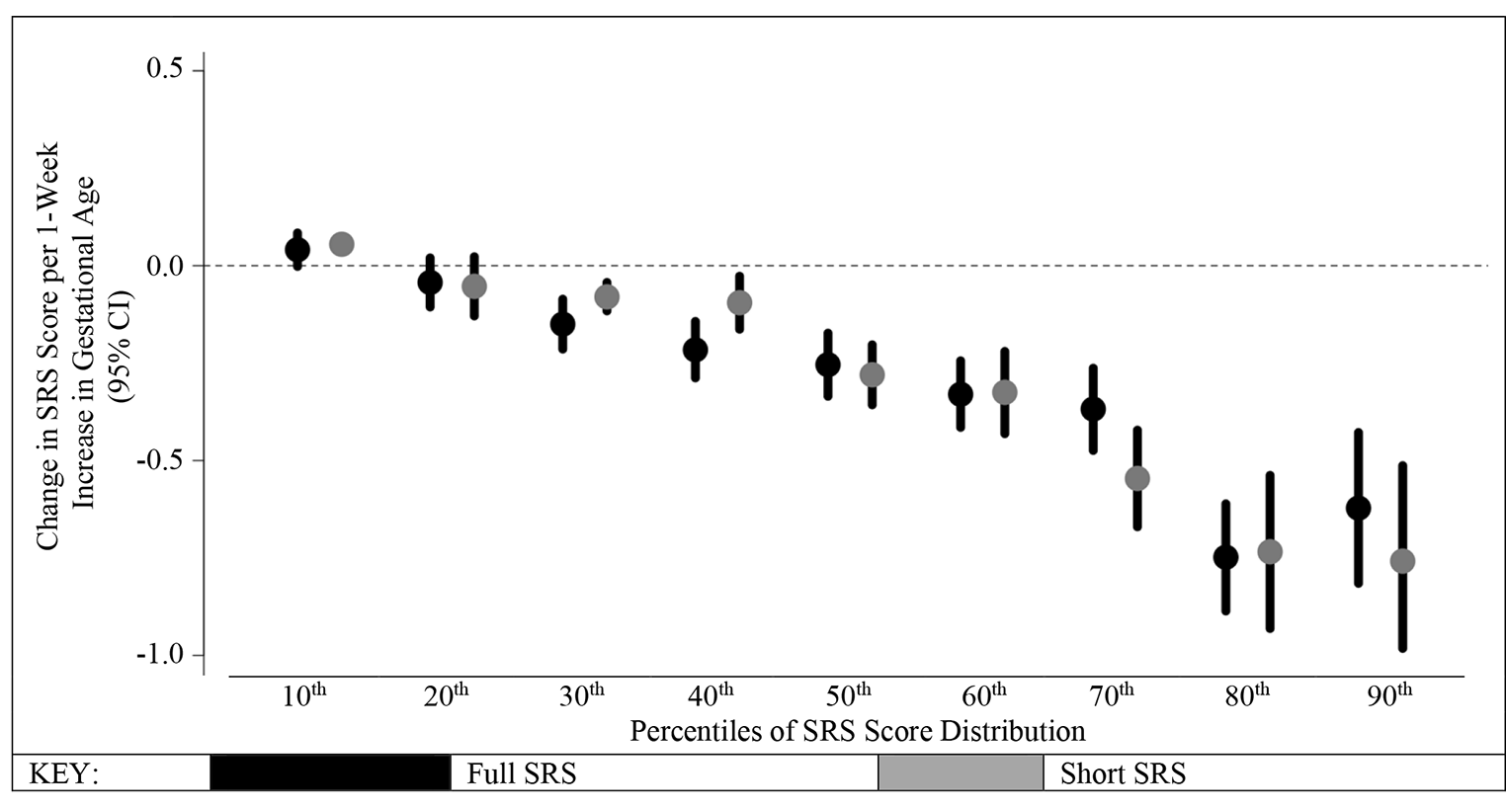
Adjusted differences in child full and short SRS scores per 1-week increase in gestational age among ECHO Cohorts (n = 2,714). ECHO = Environmental Influences on Child Health Outcomes; ASD = Autism Spectrum Disorder; ASD-ER = ASD Enriched Risk; SRS = Social Responsiveness Scale. Effect measures obtained using full SRS scores are in black, while those using short SRS scores are in gray. Models adjusted for maternal education, maternal race/ethnicity, child sex, and age at the time of SRS administration. Y-axis represents the change in SRS score (adjusted difference in SRS score) per 1-week increase in gestational age. Raw SRS scores were scaled from 0–100 to allow for comparison on the same scale. Note, values here correspond to results presented in supplementary tables S2

In quantile regression analyses, we observed similar direction and magnitude of associations with gestational age across percentiles of both the full and short SRS distribution (Fig. 1, Supplementary Table S2). These analyses also revealed increasingly stronger, inverse associations between gestational age and SRS scores with increasing SRS quantile, from the 50th (Full SRS, $\beta$: -0.3, 95% CI: -0.3, -0.2; Short SRS, $\beta$: -0.3, 95% CI: -0.4, -0.2) to the 90th (Full SRS, $\beta$: -0.6, 95% CI: -0.8, -0.4; Short SRS, $\beta$: -0.8, 95% CI: -1.0, -0.5) percentiles of the SRS score distribution. While there were some modest differences in exact estimates across quantiles between full and short scores (such as estimates closer to the null in the low-to-mid distribution of quantiles with short scores, and somewhat more attenuated estimates at the upper end of the distribution with full scores), overall, the ability to detect the association, and the magnitude of the association across quantiles was comparable.

When examining associations between preterm birth and SRS scores as the predictor (with models constructed in this fashion to address the ability of the SRS full and short scores, as potential indices of a heritable trait, to predict a known risk factor for the outcome they are meant to capture) comparable positive relative risks (RRs) were observed for both scores (Supplementary Table S3). Pre-term birth was related to continuous SRS scores in a similar magnitude for the full (RR: 1.02; 95% CI: 1.01, 1.03) and short SRS (RR: 1.01; 95% CI: 1.01, 1.02). The strength of observed associations increased when SRS scores were parameterized categorically using cut-off scores, and again, agreement across the estimates was high (full SRS RR: 1.94; 95% CI: 1.51, 2.51; short SRS RR: 1.90; 95% CI: 1.46, 2.47). We observed similar results to those seen in primary analyses when stratified by source population, suggesting comparability in estimation across different study population types.

## Discussion

Using data from the large ECHO Program, we compared the associations between gestational age, an established risk factor for ASD, with the 65-item full and 16-item short SRS. In all analyses, we observed comparable estimated effect measures between the full and short SRS, such that adjusted associations supported increases in SRS scores with preterm birth and decreases with increasing gestational age. Thus, results from these analyses support our hypothesis, and we note our objective has been achieved. Associations with gestational age were modest, though this is likely attributed to the unit (1 week), and we speculate effect sizes would be stronger with larger units. We observed a high degree of similarity in associations between gestational age with full and short SRS scores across a variety of methods, highlighting the ability of the short SRS to comparably estimate a risk factor association observed for the full SRS. Additionally, results from quantile regression analyses suggest that the inverse association between gestational age and SRS scores is increasingly stronger at higher percentiles of both the full and short SRS, suggesting a shift in the ASD-related trait distribution across the population.

The similarities in the direction and magnitude of associations observed between the full and short SRS suggest that the short SRS may be an assessment of quantitative ASD-related traits comparable to the full SRS, and that estimates of associations between a risk factor and the short SRS are comparable to those obtained using the full SRS. Thus, the short SRS may be a useful tool to abbreviate administration time of the SRS in research settings. While all psychometric properties of the short SRS have not been fully explored, advantages for its use in research settings include abbreviated administration time and lower participant burden. Evidence here suggests these gains come without compromising ability to capture risk factor associations with total SRS scores, but examination of other types of risk factors, such as genetics, is needed.

Prior work has not addressed the comparability of abbreviated quantitative trait measures like the SRS with their full counterparts for use in risk factor analyses. Previously, we confirmed comparability of the distributions of full and short SRS scores (scaled to allow for such comparisons) and prediction of ASD diagnosis in a study sample largely drawn from the general population (Lyall et al., 2021). While prior work has observed a high degree of similarity between any 16-item subset of the SRS in predicting ASD (Lyall et al., 2021) and high correlations among 18-item shortened and full SRS scores (Blanken et al., 2015; Duku et al., 2013; Román et al., 2013), as would suggest translation of findings here to other shortened versions, future work is needed to further examine compatibility in risk factor estimation across other abbreviated versions of the SRS.

A major strength of this study is the use of the large sample available through the ECHO Program. However, several limitations should be considered. First, we compared associations with just one known risk factor for ASD, gestational age, between the full and short SRS, and it is possible comparability could differ for other classes of risk factors. Second, the age of SRS administration varied (2.5–<18 years of age). While the full SRS has high reproducibility across these ages (Constantino et al., 2003; Constantino JN, Gruber C. 2012; Stickley et al., 2017), and there is considerable overlap between the preschool and school age SRS forms, future work should examine potential differences by age and short scores derived from preschool vs. school age forms, including evaluating reproducibility and test-re-test reliability. Third, ECHO cohorts with participants drawn from the general population are not necessarily generalizable to the U.S. general population, and cohorts have varying backgrounds. We did not see large differences in results when stratifying by cohort type. However, sample sizes were reduced for assessment of ASD familial cohorts, and future work should further assess the role of different study populations, and background risk, on comparability of risk factor estimation. While differences in full SRS scores did not vary by race or ethnicity in SRS standardization samples (Constantino JN, Gruber C. 2012), there have been reported differences by maternal education and family income (Moody et al., 2017); these and other demographic characteristics may be considered in future studies of the short SRS. Fourth, we were also limited by sample size to conduct other stratified analyses, including by child sex. Fifth, given limitations in data available, we could not make comparisons to other measures of ASD (Nguyen et al., 2019), nor examine the potential influence of related diagnoses on findings (Moul et al., 2015), nor were we able to compare associations across other shortened measures of the ASD-related phenotype (Sharp et al., 2023).

## Conclusions

The 65-item full SRS is commonly used as an assessment of quantitative ASD-related traits within the population and has been previously used to estimate associations with ASD risk factors. However, in studies with extensive participant follow-up, administration time may prohibit collection of longer measures, and thus, abbreviated versions are of interest. Leveraging existing data from the ECHO Program, this work adds to the growing evidence from prior research findings suggesting the comparability of this 16-item abbreviated version of the SRS to the full SRS as a quantitative trait measure of ASD. We observed consistency in estimated associations between a previously identified risk factor for ASD (gestational age) with both the full and short SRS, suggesting promise for the use of the 16-item abbreviated measure in epidemiologic risk factor analyses.

## Electronic supplementary material

Supplementary table for Tables (PDF 38 kb)

## Data Availability

De-identified data from the ECHO Program are available through NICHD’s Data and Specimen Hub (DASH). DASH is a centralized resource that allows researchers to access data from various studies via a controlled-access mechanism. Researchers can now request access to these data by creating a DASH account and submitting a Data Request Form. The NICHD DASH Data Access Committee will review the request and provide a response in approximately two to three weeks. Once granted access, researchers will be able to use the data for three years. See the DASH Tutorial for more detailed information on the process.

## References

[CR1] Beyerlein, A. (2014). Quantile regression-opportunities and challenges from a user’s perspective. *American Journal of Epidemiology*, *180*(3), 330–331. 10.1093/aje/kwu178. 24989240 10.1093/aje/kwu178

[CR2] Billeci, L., Calderoni, S., Conti, E., Gesi, C., Carmassi, C., Dell’Osso, L., et al. (2016). The broad autism (endo)phenotype: Neurostructural and Neurofunctional correlates in parents of individuals with Autism Spectrum Disorders. *Frontiers in Neuroscience*, *10*, 346. 10.3389/fnins.2016.00346. 27499732 10.3389/fnins.2016.00346 PMC4956643

[CR3] Blanken, L. M. E., Mous, S. E., Ghassabian, A., Muetzel, R. L., Schoemaker, N. K., Marroun, E., H., et al. (2015). Cortical morphology in 6- to 10-year old children with autistic traits: A population-based neuroimaging study. *The American Journal of Psychiatry*, *172*(5), 479–486. 10.1176/appi.ajp.2014.14040482. 25585034 10.1176/appi.ajp.2014.14040482

[CR4] Bölte, S., Westerwald, E., Holtmann, M., Freitag, C., & Poustka, F. (2011). Autistic traits and autism spectrum disorders: The clinical validity of two measures presuming a continuum of social communication skills. *Journal of Autism and Developmental Disorders*, *41*(1), 66–72. 10.1007/s10803-010-1024-9. 20422277 10.1007/s10803-010-1024-9 PMC3005113

[CR5] Cohen, P., Cohen, J., Aiken, L. S., & West, S. G. (1999). The problem of units and the circumstance for POMP. *Multivariate Behavioral Research*, *34*(3), 315–346. 10.1207/S15327906MBR3403_2.

[CR10] Constantino, J. N. (2012). In C. Gruber (Ed.), *Social Responsiveness Scale, Second Edition*. Los Angeles, CA: Western Psychological Services.

[CR6] Constantino, J., & Gruber, J. (2005). *Social Responsiveness Scale (SRS) Manual*. Los Angeles: Western Psychological Services.

[CR8] Constantino, J. N., & Todd, R. D. (2003). Autistic traits in the general population: A twin study. *Archives of General Psychiatry*, *60*(5), 524–530. 10.1001/archpsyc.60.5.524. 12742874 10.1001/archpsyc.60.5.524

[CR9] Constantino, J. N., & Todd, R. D. (2005). Intergenerational transmission of subthreshold autistic traits in the general population. *Biological Psychiatry*, *57*(6), 655–660. 10.1016/j.biopsych.2004.12.014. 15780853 10.1016/j.biopsych.2004.12.014

[CR7] Constantino, J. N., Davis, S. A., Todd, R. D., Schindler, M. K., Gross, M. M., Brophy, S. L., et al. (2003). Validation of a brief quantitative measure of autistic traits: Comparison of the social responsiveness scale with the autism diagnostic interview-revised. *Journal of Autism and Developmental Disorders*, *33*(4), 427–433. 10.1023/a:1025014929212. 12959421 10.1023/a:1025014929212

[CR11] Duku, E., Vaillancourt, T., Szatmari, P., Georgiades, S., Zwaigenbaum, L., Smith, I. M., et al. (2013). Investigating the measurement properties of the social responsiveness scale in preschool children with autism spectrum disorders. *Journal of Autism and Developmental Disorders*, *43*(4), 860–868. 10.1007/s10803-012-1627-4. 22915306 10.1007/s10803-012-1627-4

[CR12] Gardener, H., Spiegelman, D., & Buka, S. I. (2011). Perinatal and neonatal risk factors for autism: A comprehensive meta-analysis. *Pediatrics*, *128*(2), 10.1542/peds.2010-1036. 10.1542/peds.2010-1036 PMC3387855 21746727

[CR13] Gillman, M. W., & Blaisdell, C. J. (2018). Environmental influences on Child Health Outcomes, a Research Program of the National Institutes of Health. *Current Opinion in Pediatrics*, *30*(2), 260–262. 10.1097/MOP.0000000000000600. 29356702 10.1097/MOP.0000000000000600 PMC6020137

[CR14] Hofman, A., Jaddoe, V. W. V., Mackenbach, J. P., Moll, H. A., Snijders, R. F. M., Steegers, E. A. P., et al. (2004). Growth, development and health from early fetal life until young adulthood: The Generation R Study. *Paediatric and Perinatal Epidemiology*, *18*(1), 61–72. 10.1111/j.1365-3016.2003.00521.x. 14738548 10.1111/j.1365-3016.2003.00521.x

[CR15] Jenabi, E., Bashirian, S., Asali, Z., & Seyedi, M. (2021). Association between small for gestational age and risk of autism spectrum disorders: A meta-analysis. *Clinical and Experimental Pediatrics*, *64*(10), 538–542. 10.3345/cep.2020.01956. 33539699 10.3345/cep.2020.01956 PMC8498018

[CR16] Kaat, A. J., Croen, L. A., Constantino, J., Newshaffer, C. J., & Lyall, K. (2023). Modifying the social responsiveness scale for adaptive administration. *Quality of Life Research: An International Journal of Quality of Life Aspects of Treatment Care and Rehabilitation*. 10.1007/s11136-023-03397-y. 36943606 10.1007/s11136-023-03397-y PMC11034771

[CR17] Koenker, R. (2005). *Quantile regression*. Cambridge; New York: Cambridge University Press. 10.1017/CBO9780511754098. Accessed 13 October 2020.

[CR18] Koenker, R., & Hallock, K. F. (2001). Quantile regression. *Journal of Economic Perspectives*, *15*(4), 143–156. 10.1257/jep.15.4.143.

[CR19] Kuzniewicz, M. W., Wi, S., Qian, Y., Walsh, E. M., Armstrong, M. A., & Croen, L. A. (2014). Prevalence and neonatal factors associated with autism spectrum disorders in preterm infants. *The Journal of Pediatrics*, *164*(1), 20–25. 10.1016/j.jpeds.2013.09.021. 24161222 10.1016/j.jpeds.2013.09.021

[CR20] LeWinn, K. Z., Caretta, E., Davis, A., Anderson, A. L., & Oken, E. (2021). SPR perspectives: Environmental influences on Child Health Outcomes (ECHO) program: Overcoming challenges to generate engaged, multidisciplinary science. *Pediatric Research*, 1–8. 10.1038/s41390-021-01598-0. 10.1038/s41390-021-01598-0 PMC8204620 34131290

[CR21] Lyall, K., Hosseini, M., Ladd-Acosta, C., Ning, X., Catellier, D., Constantino, J. N., et al. (2021). Distributional Properties and Criterion Validity of a shortened version of the Social Responsiveness Scale: Results from the ECHO Program and Implications for Social Communication Research. *Journal of Autism and Developmental Disorders*, *51*(7), 2241–2253. 10.1007/s10803-020-04667-1. 32944847 10.1007/s10803-020-04667-1 PMC7965796

[CR22] Lyall, K., Rando, J., Toroni, B., Ezeh, T., Constantino, J. N., Croen, L. A., et al. (2022). Examining shortened versions of the Social Responsiveness Scale for use in autism spectrum disorder prediction and as a quantitative trait measure: Results from a validation study of 3–5 year old children. *JCPP Advances*, *2*(4), e12106. 10.1002/jcv2.12106. 36741204 10.1002/jcv2.12106 PMC9890399

[CR23] Maenner, M. J., Shaw, K. A., Bakian, A. V., Bilder, D. A., Durkin, M. S., Esler, A., & United States. (2021). Prevalence and Characteristics of Autism Spectrum Disorder Among Children Aged 8 Years - Autism and Developmental Disabilities Monitoring Network, 11 Sites, 2018. *Morbidity and Mortality Weekly Report. Surveillance Summaries (Washington, D.C.: 2002)*, *70*(11), 1–16. 10.15585/mmwr.ss7011a1 10.15585/mmwr.ss7011a1 PMC8639024 34855725

[CR24] Mahoney, A. D., Minter, B., Burch, K., & Stapel-Wax, J. (2013). Autism spectrum disorders and prematurity: A review across gestational age subgroups. *Advances in Neonatal Care: Official Journal of the National Association of Neonatal Nurses*, *13*(4), 247–251. 10.1097/ANC.0b013e31828d02a1. 23912016 10.1097/ANC.0b013e31828d02a1

[CR25] Moody, E. J., Reyes, N., Ledbetter, C., Wiggins, L., DiGuiseppi, C., Alexander, A., et al. (2017). Screening for autism with the SRS and SCQ: Variations across demographic, developmental and behavioral factors in Preschool Children. *Journal of Autism and Developmental Disorders*, *47*(11), 3550–3561. 10.1007/s10803-017-3255-5. 28856480 10.1007/s10803-017-3255-5 PMC5743015

[CR26] Moul, C., Cauchi, A., Hawes, D. J., Brennan, J., & Dadds, M. R. (2015). Differentiating autism spectrum disorder and overlapping psychopathology with a brief version of the social responsiveness scale. *Child Psychiatry and Human Development*, *46*(1), 108–117. 10.1007/s10578-014-0456-4. 24604214 10.1007/s10578-014-0456-4

[CR27] Nguyen, P. H., Ocansey, M. E., Miller, M., Le, D. T. K., Schmidt, R. J., & Prado, E. L. (2019). The reliability and validity of the social responsiveness scale to measure autism symptomology in vietnamese children. *Autism Research: Official Journal of the International Society for Autism Research*, *12*(11), 1706–1718. 10.1002/aur.2179. 31355545 10.1002/aur.2179 PMC7397486

[CR28] Robinson, E. B., Pourcain, S., Anttila, B., Kosmicki, V., Bulik-Sullivan, J. A., Grove, B., J., et al. (2016). Genetic risk for autism spectrum disorders and neuropsychiatric variation in the general population. *Nature Genetics*, *48*(5), 552–555. 10.1038/ng.3529. 26998691 10.1038/ng.3529 PMC4986048

[CR29] Román, G. C., Ghassabian, A., Bongers-Schokking, J. J., Jaddoe, V. W. V., Hofman, A., de Rijke, Y. B., et al. (2013). Association of gestational maternal hypothyroxinemia and increased autism risk. *Annals of Neurology*, *74*(5), 733–742. 10.1002/ana.23976. 23943579 10.1002/ana.23976

[CR30] Sharp, T. H., Elsabbagh, M., Pickles, A., & Bedford, R. (2023). The subcortical correlates of autistic traits in school-age children: A population-based neuroimaging study. *Molecular Autism*, *14*(1), 6. 10.1186/s13229-023-00538-5. 36765403 10.1186/s13229-023-00538-5 PMC9921646

[CR31] Stickley, A., Tachibana, Y., Hashimoto, K., Haraguchi, H., Miyake, A., Morokuma, S., et al. (2017). Assessment of autistic traits in children aged 2 to 4½ years with the Preschool Version of the Social Responsiveness Scale (SRS-P): Findings from Japan. *Autism Research*, *10*(5), 852–865. 10.1002/aur.1742. 28256099 10.1002/aur.1742 PMC6586029

[CR32] Sturm, A., Kuhfeld, M., Kasari, C., & McCracken, J. T. (2017). Development and validation of an item response theory-based Social Responsiveness Scale short form. *Journal of Child Psychology and Psychiatry and Allied Disciplines*, *58*(9), 1053–1061. 10.1111/jcpp.12731. 28464350 10.1111/jcpp.12731

[CR33] Volkow, N. D., Koob, G. F., Croyle, R. T., Bianchi, D. W., Gordon, J. A., Koroshetz, W. J., et al. (2018). The conception of the ABCD study: From substance use to a broad NIH collaboration. *Developmental Cognitive Neuroscience*, *32*, 4–7. 10.1016/j.dcn.2017.10.002. 29051027 10.1016/j.dcn.2017.10.002 PMC5893417

