# Supplementary material for: A Comparative Analysis of the Full and Short Versions of the Social Responsiveness Scale in Estimating an Established Autism Risk Factor Association in ECHO: Do we Get the Same Estimates?
Source: J Autism Dev Disord. 2023 Jul 22;55(6):2050–8. doi: 10.1007/s10803-023-06020-8 (PMC11370280; doi:10.1007/s10803-023-06020-8)
Supplement: Supplementary file 1 — Supplementary table for Tables (PDF 38 kb) [file 10803_2023_6020_MOESM1_ESM.docx]

**SUPPLEMENTARY MATERIAL**

**A Comparative Analysis of the Full and Short Versions of the Social Responsiveness Scale in Estimating an Established Autism Risk Factor Association in ECHO: Do We Get the Same Estimates?**

***Journal of Autism and Developmental Disorders***

Marisa A. Patti^1^, Xuejuan Ning^2,3^, Mina Hosseini^2,3^, Lisa A. Croen^4^, Robert M. Joseph^5^, Margaret R. Karagas^6^, Christine M. Ladd-Acosta^2,3^, Rebecca Landa^7^, Daniel S. Messinger^8^, Craig J. Newschaffer^1,9^, Ruby Nguyen^10^, Sally Ozonoff^11^, T. Michael O’Shea^12^, Rebecca J. Schmidt^13^_,_ Cindy O. Trevino^14^, Kristen Lyall^1^, on behalf of program collaborators for the Environmental Influences on Child Health Outcomes*

*See Acknowledgments for full listing of collaborators

^1^A.J. Drexel Autism Institute, Drexel University, Philadelphia, PA, USA; ^2^ Department of Epidemiology, Johns Hopkins Bloomberg School of Public Health, Baltimore, MD, USA;

^3^Wendy Klag Center for Autism and Developmental Disabilities, Johns Hopkins Bloomberg School of Public Health, Baltimore, MD, USA; ^4^Division of Research, Kaiser Permanente, Oakland, CA, USA; ^5^Department of Anatomy and Neurobiology, Boston University School of Medicine, Boston, MA, USA; ^6^Department of Epidemiology, Geisel School of Medicine, Dartmouth College, Hanover, NH, USA; ^7^Center for Autism and Related Disorders, Kennedy Krieger Institute; Department of Psychiatry and Behavioral Sciences, Johns Hopkins University School of Medicine, Baltimore, MD, USA; ^8^Departments of Psychology and Pediatrics, University of Miami, Coral Gables, FL, USA; ^9^College of Health and Human Development, Pennsylvania State University, University Park, PA, USA; ^10^Department of Epidemiology and Community Health, University of Minnesota, Minneapolis, MN, USA; ^11^Department of Psychiatry and Behavioral Sciences, MIND Institute, University of California Davis, Sacramento, CA, USA; ^12^ Department of Pediatrics, University of North Carolina School of Medicine, Chapel Hill, NC, USA; ^13^Department of Public Health Sciences, UC Davis, Davis CA and the UC Davis MIND Institute, Sacramento, CA, USA; ^14^Department of Psychiatry and Behavioral Sciences, University of Washington, Seattle Children’s Research Institute, Seattle, WA, USA.

**Corresponding Author:**

Marisa A. Patti

A.J. Drexel Autism Institute

Drexel University

Email: map534@drexel.edu

| TABLE OF CONTENTS | Page # |
| --- | --- |
| **Table S1** Associations between gestational age and preterm birth with child SRS scores using the full and short SRS ……………………………………………………. | 3 |
| **Table S2** Adjusted differences in child full and short SRS scores per 1-week increase in gestational age ………………………………………………………………………. | 4 |
| **Table S3** Ability for the full and short SRS to predict preterm birth …………………. | 5 |

| **Table S1** Associations between gestational age and preterm birth with child SRS scores^a^ using the full and short SRS among ECHO Cohorts | | | | | | | | | | | |
| --- | --- | --- | --- | --- | --- | --- | --- | --- | --- | --- | --- |
|  |  | Full SRS | | | | | |  | Short SRS | | |
|  |  | Crude |  | | Adjusted^c^ | |  | | Crude |  | Adjusted |
|  | N^b^ | $\beta$ (95% CI) | | $\beta$ (95% CI) | | | | | $\beta$ (95% CI) | | $\beta$ (95% CI) |
| Continuous GA^d^ |  |  | | | |  | | |  | |  |
| Total sample | 2714 | -0.5 (-0.6, -0.4) | | | | -0.3 (-0.4, -0.2) | | | -0.5 (-0.6, -0.4) | | -0.3 (-0.4, -0.2) |
| General cohorts^e^ | 1985 | -0.2 (-0.5, 0.0) | | | | -0.2 (-0.4, 0.1) | | | -0.1 (-0.2, 0.1) | | -0.1 (-0.2, 0.1) |
| Familial ASD enriched cohorts^f^ | 296 | -1.2 (-2.1, -0.3) | | | | -1.1 (-2.0, -0.3) | | | -0.5 (-1.0, 0.0) | | -0.5 (-1.0, 0.0) |
| Preterm birth cohorts^g^ | 433 | -2.2 (-3.4, -1.0) | | | | -1.7 (-2.9, -0.5) | | | -1.4 (-2.0, -0.7) | | -1.2 (-1.8, -0.5) |
| Preterm birth^h^ |  |  | | | |  | | |  | |  |
| Total sample | 2714 | 5.1 (4.0, 6.2) | | | | 2.8 (1.7, 4.0) | | | 5.3 (4.0, 6.5) | | 2.9 (1.6, 4.3) |
| General cohorts | 1985 | 1.3 (-0.4, 3.1) | | | | 1.1 (-0.5, 2.8) | | | 0.5 (-0.6, 1.5) | | 0.4 (-0.6, 1.4) |
| Familial ASD enriched cohorts | 296 | 4.5 (-4.0, 13.1) | | | | 4.8 (-3.7, 13.4) | | | 1.0 (-3.7, 5.8) | | 1.6 (-3.2, 6.5) |
| ECHO = Environmental influences on Child Health Outcomes; ASD = Autism Spectrum Disorder; SRS = Social Responsiveness Scale; GA = Gestational Age.  ^a^Raw SRS scores were scaled from 0-100 to allow for comparison on the same scale.  ^b^Refers to the number of participants in analysis.  ^c^Adjusted for maternal education, maternal race/ethnicity, child sex, age at SRS administration.  ^d^Continuous gestational age modeled as 1-week increase in gestational age.  ^e^General population refers to ECHO cohorts drawn from the general population.  ^f^Familial ASD Enriched Cohorts refers to ECHO cohorts with participants at high familial risk for ASD, due to selective enrollment of children whose mothers previously had a child diagnosed with ASD.  ^g^Preterm birth cohorts refer to selective enrollment of children born preterm.  ^h^Preterm birth is defined as <37 weeks gestation. | | | | | | | | | | | |

| **Table S2** Adjusted differences in child full and short SRS scores^a^ per 1-week increase in gestational age among ECHO cohorts | | | |
| --- | --- | --- | --- |
|  | Gestational age^b^ | | |
|  | Full SRS |  | Short SRS |
| Percentile | $\beta$ (95% CI)^c^ | | $\beta$ (95% CI) |
| 10 | 0.0 (0.0, 0.1) | | 0.1 (0.0, 0.1) |
| 20 | 0.0 (-0.1, 0.0) | | -0.1 (-0.1, 0.0) |
| 30 | -0.2 (-0.2, -0.1) | | -0.1 (-0.1, 0.0) |
| 40 | -0.2 (-0.3, -0.1) | | -0.1 (-0.2, 0.0) |
| 50 | -0.3 (-0.3, -0.2) | | -0.3 (-0.4, -0.2) |
| 60 | -0.3 (-0.4, -0.2) | | -0.3 (-0.4, -0.2) |
| 70 | -0.4 (-0.5, -0.3) | | -0.5 (-0.7, -0.4) |
| 80 | -0.7 (-0.9, -0.6) | | -0.7 (-0.9, -0.5) |
| 90 | -0.6 (-0.8, -0.4) | | -0.8 (-1.0, -0.5) |
| ECHO = Environmental influences on Child Health Outcomes; SRS = Social Responsiveness Scale.  ^a^Raw SRS scores were scaled from 0-100 to allow for comparison on the same scale.  ^b^Adjusted for maternal education, maternal race/ethnicity, child sex, age at SRS administration.  ^c^$\beta$ values represent the change in SRS score (adjusted difference in SRS score) per 1-week increase in gestational age. | | | |

| **Table S3** Ability for the full and short SRS^a^ to predict preterm birth^b^ among ECHO cohorts | | | | | | | | | |  |
| --- | --- | --- | --- | --- | --- | --- | --- | --- | --- | --- |
|  |  | Full SRS | | | |  | Short SRS | | |  |
|  |  | Crude |  | Adjusted^d^ |  | | Crude |  | Adjusted |  |
|  | N^c^ | RR (95% CI) | | RR (95% CI) | | | RR (95% CI) | | RR (95% CI) |  |
| Continuous SRS score |  |  | |  | | |  | |  |  |
| Total sample | 2714 | 0.99 (0.96, 1.02) | | 1.02 (1.01, 1.03) | | | 1.02 (1.02, 1.03) | | 1.01 (1.01, 1.02) | |
| General population cohorts^e^ | 1985 | 1.01 (1.00, 1.01) | | 1.01 (1.00, 1.01) | | | 1.01 (0.99, 1.02) | | 1.01 (0.99, 1.02) | |
| Familial ASD enriched cohorts^f^ | 296 | 1.01 (0.99, 1.04) | | 1.02 (0.99, 1.06)* | | | 1.01 (0.97, 1.05) | | 1.03 (0.97, 1.09)* | |
| Cut-off SRS Scores^g^ |  |  | |  | | |  | |  | |
| Total Sample | 2714 | 2.98 (2.39, 3.73) | | 1.94 (1.51, 2.51) | | | 2.68 (2.13, 3.38) | | 1.90 (1.46, 2.47) | |
| General population cohorts | 1985 | 1.19 (0.74, 1.93) | | 1.19 (0.72, 1.97) | | | 1.19 (0.73, 1.94) | | 1.23 (0.74, 2.05) | |
| Familial ASD enriched cohorts^h^ | 296 | -- | | -- | | | -- | | -- | |
| ECHO = Environmental influences on Child Health Outcomes; ASD = Autism Spectrum Disorder; SRS = Social Responsiveness Scale.  ^a^Raw SRS scores were scaled from 0-100 to allow for comparison on the same scale.  ^b^Preterm birth defined as <37 weeks gestation.  ^c^Refers to the number of participants in analysis.  ^d^Adjusted for maternal education, maternal race/ethnicity, child sex, age at SRS administration.  ^e^General population refers to ECHO cohorts drawn from the general population.  ^f^Familial ASD enriched refers to ECHO cohorts with participants at high familial risk for ASD, due to selective enrollment of children whose mothers previously had a child diagnosed with ASD.  ^g^Cut-off scores defined ass 52 for the full SRS, 13 for the short SRS, 27 for uniform scaled SRS.  ^h^Could not be estimated reliably due to small sample size.  * 0 preterm case in 4^th^ maternal race group, results in quasi-separation. | | | | | | | | | | |
